# A map of mobile DNA insertions in the NCI-60 human cancer cell panel

**DOI:** 10.1186/s13100-016-0078-4

**Published:** 2016-10-31

**Authors:** John G. Zampella, Nemanja Rodić, Wan Rou Yang, Cheng Ran Lisa Huang, Jane Welch, Veena P. Gnanakkan, Toby C. Cornish, Jef D. Boeke, Kathleen H. Burns

**Affiliations:** 1 Department of Dermatology, Johns Hopkins University School of Medicine, 733 North Broadway, Miller Research Building Room 469, Baltimore, MD 21205 USA; 2 Department of Pathology, Johns Hopkins University School of Medicine, 733 North Broadway, Miller Research Building Room 469, Baltimore, MD 21205 USA; 3 McKusick-Nathans Institute of Genetic Medicine, 733 North Broadway, Miller Research Building Room 469, Baltimore, MD 21205 USA; 4 High Throughput (HiT) Biology Center, 733 North Broadway, Miller Research Building Room 469, Baltimore, MD 21205 USA; 5 The Sidney Kimmel Comprehensive Cancer Center, Johns Hopkins University School of Medicine, 733 North Broadway, Miller Research Building Room 469, Baltimore, MD 21205 USA; 6 Present address: Institute for Systems Genetics, NYU Langone University School of Medicine, New York, NY 10016 USA

## Abstract

**Background:**

The National Cancer Institute-60 (NCI-60) cell lines are among the most widely used models of human cancer. They provide a platform to integrate DNA sequence information, epigenetic data, RNA and protein expression, and pharmacologic susceptibilities in studies of cancer cell biology. Genome-wide studies of the complete panel have included exome sequencing, karyotyping, and copy number analyses but have not targeted repetitive sequences. Interspersed repeats derived from mobile DNAs are a significant source of heritable genetic variation, and insertions of active elements can occur somatically in malignancy.

**Method:**

We used Transposon Insertion Profiling by microarray (TIP-chip) to map Long INterspersed Element-1 (LINE-1, L1) and *Alu* Short INterspersed Element (SINE) insertions in cancer genes in NCI-60 cells. We focused this discovery effort on annotated Cancer Gene Index loci.

**Results:**

We catalogued a total of 749 and 2,100 loci corresponding to candidate LINE-1 and *Alu* insertion sites, respectively. As expected, these numbers encompass previously known insertions, polymorphisms shared in unrelated tumor cell lines, as well as unique, potentially tumor-specific insertions. We also conducted association analyses relating individual insertions to a variety of cellular phenotypes.

**Conclusions:**

These data provide a resource for investigators with interests in specific cancer gene loci or mobile element insertion effects more broadly. Our data underscore that significant genetic variation in cancer genomes is owed to LINE-1 and *Alu* retrotransposons. Our findings also indicate that as large numbers of cancer genomes become available, it will be possible to associate individual transposable element insertion variants with molecular and phenotypic features of these malignancies.

**Electronic supplementary material:**

The online version of this article (doi:10.1186/s13100-016-0078-4) contains supplementary material, which is available to authorized users.

## Significance statement

Transposable elements are repetitive sequences that comprise much of our DNA. They create both inherited and somatically acquired structural variants. Here, we describe a first generation map of LINE-1 and *Alu* insertions in NCI-60 cancer cell lines. This provides a resource for discovering and testing functional consequences of these sequences.

## Background

The National Cancer Institute-60 (NCI-60) cell panel was developed in the 1980s as a tool for pharmacologic screens and has become the most extensively studied collection of human cancers [1]. The panel comprises 59 cell lines encompassing nine tissue origins of malignancy, including blood, breast, colon, central nervous system, kidney, lung, ovary, prostate, and skin [2]. They have become a resource for high throughput characterizations and systems biology based approaches to cancer.

NCI-60 cell genomes have been described by targeted [3] and whole exome sequencing [4], karyotyping [5], and assays to detect copy number alteration [6], loss of heterozygosity [7], and DNA methylation [8]. Large scale mRNA [9] and microRNA [10] expression, protein abundance [11] and phosphorylation [12], and metabolomic [13] studies have also been conducted. Because assays are applied across the panel of cell lines in each case, datasets from orthogonal studies can be related to one another. For example, gene expression patterns have been found to be predictive of chemotherapeutic sensitivities [9].

Interspersed repeats have not been incorporated in these or many other genome-wide surveys. These repetitive sequences are dynamic constituents of human genomes and important sources of structural variation [14–20]. RNA transcribed from active elements can be reverse transcribed and integrated into the genome at new sites by proteins encoded by LINE-1 (Long INterspersed Element)-1 [21–23]. The result is that relatively recent insertions of LINE-1 (L1Hs) and *Alu* SINEs (*Alu*Ya5, *Alu*Ya8, *Alu*Yb8, *Alu*Yb9) are sources of genetic polymorphisms where both the pre-insertion allele and the insertion allele coexist in human populations. Moreover, LINE-1 sequences are hypomethylated [24–28] and express protein in a wide variety of human cancers [29], and somatic LINE-1 integrations have been reported in tumor genomes [15, 30–36].

It is well established that inherited and acquired mobile DNA insertions can affect gene expression; there is inherent potential for insertions to have effects on tumor biology. However, the large majority occur in intronic or intergenic regions. Strong biases in the distribution of insertion sites or recurrent ‘hotspots’ for insertions arising during tumor development are frequently not obvious, leading to the presumption that most are non-functional ‘passenger mutations’ [34, 36].

This is *not* such a tumor-normal comparison study, but rather, one aimed to identify potential functions of mobile DNAs in human cancer cells. Towards this end, we mapped LINE-1 and *Alu* insertions in the NCI-60 tumor cell panel. We used a method for interspersed repeat mapping, Transposon Insertion Profiling by microarray (TIP-chip), to identify insertion sites. We also use previous characterizations of the cell panel to associate specific insertions with cellular phenotypes.

## Results

### Transposon insertion profiling by microarray

**Fig. 1:**
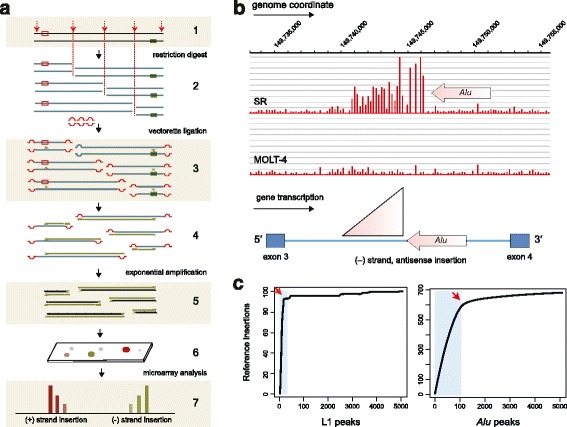
Mapping transposable element (TE) insertion sites. **a**. A schematic illustrating the sequential steps of Transposon Insertion Profiling by microarray (TIP-chip). (1) An interval of double stranded genomic DNA with two TE insertions (boxes) oriented on opposing strands is shown; (2) the DNA is digested in parallel restriction enzyme reactions and ligated to vectorette oligonucleotides; (3) oligonucleotides complementary to the TE insertions prime first strand synthesis; (4) the elongating strands form reverse complements of the vectorette sequence; (5) there is exponential amplification of insertion site fragments; (6) these amplicons are labeled and hybridized to genomic tiling microarrays; and (7) ‘peaks’ of fluorescence intensity across several probes corresponding to contiguous genomic positions indicate a TE insertion. **b**. An example of a polymorphic *Alu* peak in two leukemia cell lines (SR and MOLT-4) in the third intron of the *TCOF1* (Treacher Collins-Franceschetti syndrome 1) gene on chromosome 5. The upper panels show TIP-chip data for the insertion, which is present in the SR line and not the MOLT-4 cells. The *Alu* insertion is a minus (-) strand insertion to the right of the probe with the greatest intensity; an arrow is drawn to indicate its position and orientation, but the arrow is not drawn to scale. *Alu* insertions approximate 300 bp, and the width of the peak in this case is 5 kb. **c**. Peaks were recognized using a sliding window algorithm which identified adjacent probes above a threshold fluorescence intensity value. The threshold value was progressively lowered to identify peaks in a rank order. The graphs show the number of reference insertions identified verses peak rank for a representative LINE-1 and *Alu* TIP-chip. The cut-off for defining a candidate insertion was established using the inflection points (red arrows) of these plots

To map mobile DNA insertions, we used a method we have termed transposon insertion profiling by microarray (TIP-chip), which uses vectorette PCR to amplify unknown sequence adjacent to a known primer-binding site (Fig. 1a). We surveyed three major currently active mobile DNAs in humans (L1Hs, *Alu*Ya5/8; and *Alu*Yb8/9) as previously described [14]. To focus on the potential functional impact of these sequences on cancer cell phenotypes, PCR amplicons were labeled and analyzed using a genomic tiling microarray designed to encompass 6,484 known Cancer Gene Index loci (+/- 10 kb) (Biomax™ Informatics), about 17 % of the genome. Peaks of signal intensity correspond to TE insertions (Fig. 1a, b); known LINE-1 and *Alu* elements incorporated in the reference genome assembly (hereafter, ‘reference insertions’) were used as a quality control metric and to set cut-offs for recognized peaks (Fig. 1c).

**Fig. 2:**
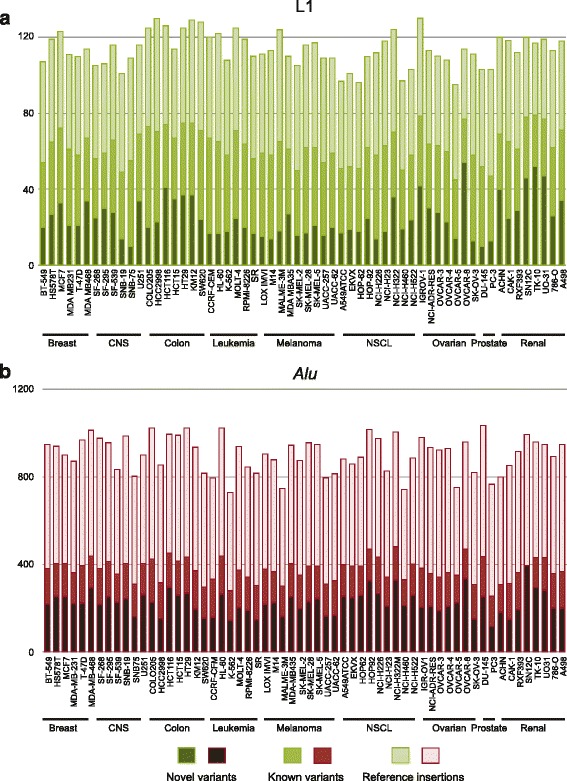
Total TE insertions. The stacked bar plots show the relative numbers of novel variants, known variants, and reference insertions per cell line for LINE-1 (green, *upper panel*) and *Alu* (red, *lower panel*). The total number of insertions detected per cell line is similar across the tumor panel

**Fig. 3:**
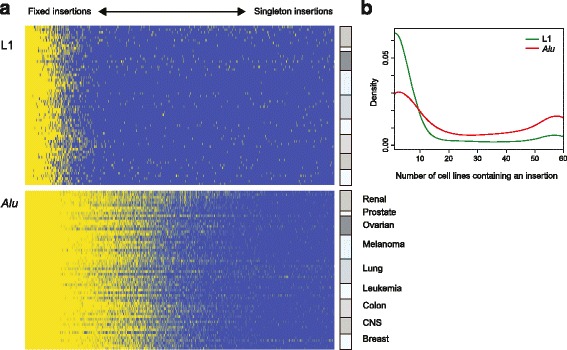
Distribution of TE insertions across the NCI-60 panel. **a**. Individual insertions are arrayed in order of frequency horizontally, and cell lines are arrayed vertically. Yellow denotes presence of insertion; blue denotes absence. LINE-1 are on the upper plot, and *Alu* are on the lower. Cell types are listed for the lower panel, and the ordering is the same in the upper panel. **b**. The density plot shows proportions of insertions against the numbers of cell lines containing an insertion. For both *Alu* (red) and LINE-1 (green), there is a bimodal distribution. The leftmost density reflects a large number of polymorphic insertions with low allele frequencies and (for LINE-1 singletons) somatically acquired insertions. The rightmost increase in density shows common variants or fixed insertions present in most or all cell lines

A total of 749 and 2,100 peaks corresponding to candidate LINE-1 and *Alu* insertion sites respectively were recognized across the NCI-60 cell panel. These locations were cross-referenced to previously described insertions to define three categories: (*i*.) reference insertions, which include invariant insertions and insertion polymorphisms incorporated in the reference genome assembly; (*ii*.) inherited variants either previously described (known polymorphic) or newly discovered, but occurring in multiple, unrelated cell lines (novel polymorphic); and (*iii*.) novel, ‘singleton’ insertions seen uniquely in one cell line (Fig. 2a, b). The last category includes both insertions that were constitutive (germline) in the patient from whom the cell line was derived as well as somatic insertions acquired during tumor development or the propagation of these cell lines. A greater proportion of LINE-1 insertions were singletons (68 %) compared with *Alu* insertions (21 %). Density plots for both LINE-1 and *Alu* show most peaks fall into this last category, particularly for L1Hs, although a biphasic distribution was seen (Fig. 3a, b).

Our array encompassed 130 known reference LINE-1 and 1278 *Alu* insertions. A total of 112 LINE-1 and 1,160 *Alu* insertions detected were present in the reference genome assembly. A total of 697 LINE-1 and 1,147 *Alu* insertions were singleton or polymorphic (known and novel) segregating in human populations (Fig. 2a, b). Insertions incorporated in the reference genome that are known to be polymorphic are counted in both groups. A summary of insertion positions by tumor type and cell line can be found in Additional file 1: Table S1, Additional file 2: Table S2.

We found that each cell line had a unique transposable element (TE) insertion profile (Fig. 3a). After correcting for batch effects, a principal component analyses (PCA) did not show clustering by tumor type. As expected, however, pairs of cell lines derived from the same individual grouped together, and these pairs showed a high concordance of top-ranking peaks as compared to unrelated cell lines. We compared TE insertion profiles to described cytogenetic abnormalities. In some instances, insertions were informative of deletions; for example, a reference LINE-1 in the retinoblastoma 1 (*RB1*) locus was only absent in the MB468 breast cancer cell line, consistent with the homozygous deletion of *RB1* reported for this cell line [37].

### Insertions in genes involved in oncogenesis

In TIP-chip, probe spacing does not resolve insertions to the precise base, and insertion strandedness was not predicted for all peak intervals in this study. Despite these limitations, we identified peak intervals that partially or entirely overlapped exon intervals for further inspection. Partial overlaps were almost entirely attributable to insertions near an exon. We identified 9 insertions within exons, and all were located within gene 3’ untranslated regions (3’ UTRs); none affected protein open reading frames.

**Fig. 4:**
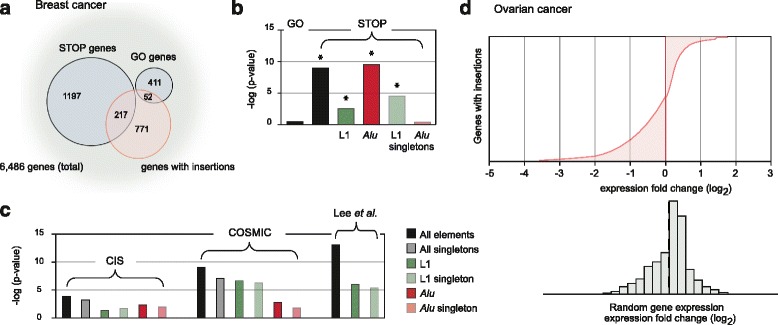
TE enrichment analyses. **a**. STOP and GO genes have been implicated in breast cancer as genes that appear to inhibit and promote tumor development, respectively. Using a hypergeometric distribution to assess enrichment, we found that TE insertions are enriched in STOP genes on the array (*p* = 1.23x10^-9^) but not in GO genes (*p* = 0.33). **b**. The bar graph shows enrichment by type of TE plotted as the negative log of the p-value. No GO gene enrichment is seen. STOP gene enrichment is seen considering all LINE-1 (*p* = 3.11x10^-3^); all *Alu* (*p* = 2.27x10^-10^); as well as LINE-1 singletons (*p* = 4.16x10^-5^). **c**. Insertions were also enriched in common insertion sites (CIS) (*p* = 1.46x10^-4^); COSMIC commonly mutated cancer genes (*p* = 7.74x10^-10^); and genes reported to acquire somatic LINE-1 insertions in cancer by Lee et al. (*p* = 5.34x10^-14^). **d**. Genes with TE insertions in ovarian cancer cell lines are more likely than other genes to be downregulated in ovarian cancer samples as compared to normal tissue controls. Randomly selected genes are shown for comparison (*bottom panel*)

To begin to approach potential functional consequences of intronic insertions, we analyzed insertion sites in sets of genes with described roles in cancer. We considered collections of genes with TE insertions while grouping together malignant cell lines by tissue of origin. Interestingly, in breast cancer cell lines, we observed a significant enrichment of singleton and polymorphic LINE-1 and *Alu* insertions in “STOP genes”, defined in shRNA screens as suppressors of human mammary epithelial cell proliferation [38] (*p* = 1.23x10^-9^) (Fig. 4a). This result persisted when LINE-1 and *Alu* insertions were analyzed independently; LINE-1 singleton insertions but not *Alu* singleton insertions were also enriched in this gene set (Fig. 4b). Analysis of expression of these “STOP” genes shows that a preponderance of these genes are down-regulated; this result persists in those genes containing a TE insertion. The findings suggest that collectively, insertions may act to compromise expression of these genes.

Consistent with this model, ovarian cancer cell lines showed a preponderance of insertions in genes that are down regulated in ovarian cancers as compared to normal tissue. A random set of genes from the array is shown as a histogram for comparison (Fig. 4d). This pattern was absent in other tumor types.

We saw an enrichment of singleton and polymorphic TEs in genes recurrently mutated in experimental cancer models and in human tumors. For the former, we considered common insertion sites (CIS) defined as gene loci recurrently interrupted by insertional mutagens in forward cancer gene screens in mice [39, 40] (*p* = 1.46x10^-4^). The latter was assessed using genes frequently mutated in human cancers taken from the Catalogue Of Somatic Mutations In Cancer (COSMIC) database [41] (*p* = 7.74x10^-10^) (Fig. 4c). We also compared our insertion profiles to sites of reported somatic TE insertions in human cancers. We analyzed novel (singleton and polymorphic) insertions and discovered that we had overlaps in 22 of the 64 genes noted by Lee et al. [32] and 23 of 76 from Solimini et al. [38](Fig. 4c). We anticipate the possibility that common insertion site loci will be identified as more insertion site mapping studies are conducted in human tumors.

### Functional associations of individual insertions

**Fig. 5:**
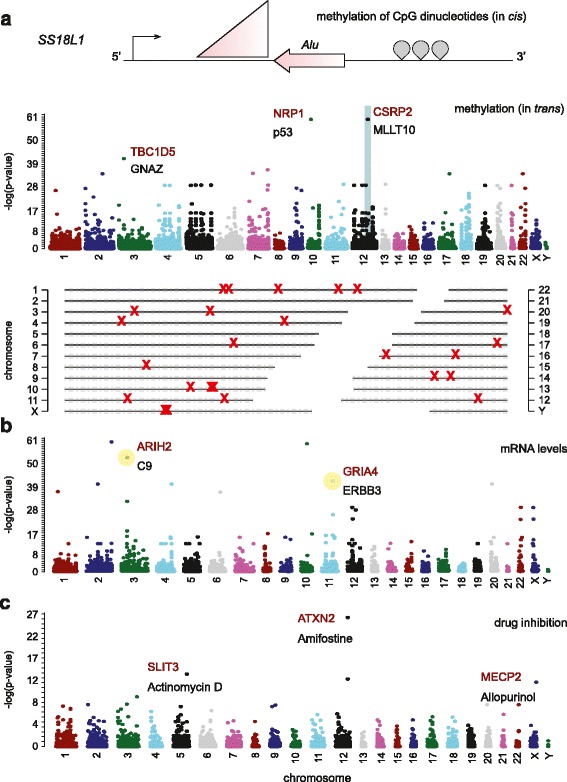
TE insertions associated with cellular phenotypes. **a**. Associations with DNA methylation. (*Upper panel*) Diagram of the *SS18L1* (Synovial sarcoma translocation gene on chromosome 18-like 1) gene locus, which contains an antisense *Alu* associated with increased CpG methylation at that gene (i.e., in *cis*, *p* = 3.67x10^-6^) (*Middle panel*) Manhattan plot showing TE positions on the x-axis and strengths of association with gene methylation on the y-axis (Bonferroni-corrected p-values). Singleton insertions were excluded from association analyses. Gene abbreviations are given for both the gene in which the insertion is found (red) and the associated methylation site (black) in examples. The TE insertion at the *CSRP2* (cysteine and glycine-rich protein 2) gene locus was associated with methylation at 22 distinct loci (*Lower panel*). The associated methylation sites are distributed throughout the genome. **b**. Manhattan plot showing associations with steady state mRNA levels. Gene abbreviations are given for both the gene in which the insertion is found (red) and the associated transcript level (black); in these two examples, the TE is associated with upregulation of the mRNA. **c**. Manhattan plot showing associations with drug sensitivity as measured by total cellular growth inhibition. The gene in which the insertion is found is given (red), as well as the associated pharmacologic agent (black)

An advantage of working with the NCI-60 cell lines is that these are well studied. To integrate our insertion site maps with other findings in these cells, we performed COMPARE analyses [42]. COMPARE is a pattern matching method developed specifically for NCI-60 cell lines that provides a *p*-value for each association (S5–25). Direct, local roles for TEs (in *cis*) were not observed for the majority of correlations. However, COMPARE did reveal three insertions associated with DNA hypermethylation within 30 kb of the insertion site. For example, a polymorphic *Alu* insertion in the *SS18L1* (Synovial sarcoma translocation gene on chromosome 18-like 1) gene locus oriented anti-sense to the transcription of the gene, is associated with increased methylation of nearby CpG sites at the same gene locus (*p* = 6.67x10^-6^) (Fig. 5a).

Manhattan plots illustrate highly significant correlations found in *trans* (Fig. 5a–c). A subset of insertions had multiple associations (vertical series of dots corresponding to one TE location), suggesting the possibility of pleomorphic effects of an insertion haplotype.

In addition, we encountered examples of single ‘driver’ mutations and cellular phenotypes that could be associated with multiple TE insertions. Five insertions correlated with a mutation in the *ERBB2* gene (v-erb-b2 erythroblastic leukemia viral oncogene homolog 2, the HER2/neu locus), and more than 10 insertions were associated with thymidylate synthase activity (*p* values < 10^-20^). To probe relationships between multiple *trans* associated factors related to a single TE insertion, we performed pathway analyses on sets of genes, each encompassing the TE insertion locus and all RNAs and proteins with associated expression patterns. This yielded more than 250 curated pathways with enrichment *p*-values less than 10^-4^, supporting the concept that these are biologically relevant as opposed to spurious associations. All COMPARE results are provided in the (Additional file 3: Table S3).

## Discussion

Our genomes are filled with highly repetitive DNA sequences derived from TEs. Tailored methods for their detection, including TIP-chip [14], targeted insertion site sequencing [15, 17, 18, 31, 36, 43], and algorithms for finding variants in whole genome sequencing [20, 34, 44] are revealing this previously masked dimension of genomic data. Collectively, these studies confirm that TEs are rich sources of genetic diversity in human populations, and provide evidence that they are somatically unstable in a variety of tumor types. Of the two most active germline elements, LINE-1 and *Alu*, (which is mobilized in *trans* by LINE-1-encoded proteins), LINE-1 has been more well documented to be active in cancer. *Alu* insertions account for more inherited polymorphisms. For both types of TEs, the vast majority of catalogued insertions are intronic and intergenic without clear function.

To begin a systematic survey for functionally consequential LINE-1 and *Alu* integrations in human neoplasias, we mapped these variants in the NCI-60 cell panel. NCI-60 is a unique resource for this, encompassing a variety of cancer cell lines that have the advantages of being well studied and readily available. We mapped LINE-1 and *Alu* insertion positions using a microarray-based approach over a large census of cancer genes. Even as TIP-chip is replaced by sequencing, we expect these data will provide a useful reference.

TIP-chip across the NCI-60 panel revealed numerous novel candidate TEs, totaling about 500 L1Hs and 1000 *AluYa/Yb* insertions distributed across the 60 cell lines. These include insertions that are unique to a cell line (‘singleton’) and novel polymorphic insertions (found in unrelated cell lines). Although ‘singletons’ may be enriched for tumor-specific, somatic insertion events, matched non-neoplastic cells for the corresponding patient cases are not available, and therefore we cannot definitively differentiate somatic from inherited variants. Similarly, these cell lines have undergone numerous passages since their creation, and somatic insertion events occurring in culture cannot be clearly recognized. We note a greater proportion of LINE-1 singletons (68 % of LINE-1 loci) than *Alu* singletons (21 % of *Alu* loci), consistent with ongoing LINE-1 retrotransposition in vivo or in vitro.

We approached the question of TE function by two avenues. We first tested for biases in the distribution of insertions with respect to known gene sets. We found a preferential accumulation of TE insertions in retained copies of ‘STOP genes’ in breast cancer cell lines; these gene loci function as inhibitors of mammary epithelial cell proliferation. Experimental models suggest that it is advantageous for tumor growth to compromise the function of these genes [38], and we speculate that TE insertions are enriched at these loci because they have a role in this process. These ‘STOP genes’ are downregulated in the breast cancer cell lines, as is the subset of ‘STOP genes’ containing TE insertions. We also found preferential TE accumulation in genes downregulated in ovarian cancers compared with normal ovarian tissue, which would be consistent with this model. Finally, genes with functional roles in cancer were also more commonly seen as insertion sites than expected. These included genes ‘hit’ recurrently by insertional mutagenesis in forward genetics screens in mice, the so-called common insertion sites (CIS), and in genes commonly mutated in human cancers (COSMIC catalog) [41].

We note that the exonizations of intronic LINE-1 [45] and *Alu* sequences [46] are being increasingly recognized using RNA-seq, and that many of the resulting transcripts have an altered protein coding capacity. It may be possible to identify aberrant mRNA species corresponding to these insertion loci and thus invoke a molecular mechanism to underlie this type of functional effect.

Our second approach relied on association studies. We used existing data in COMPARE analyses to test for relationships between TE insertion alleles and cellular phenotypes. In the case of DNA methylation only, *cis* effects could be seen relating individual TEs with local DNA hypermethylation. We identified three *Alu* integrations associated with DNA hypermethylation at the insertion site (+/- 30 kb). The most notable is a polymorphic *Alu* insertion in the first intron of the *SS18L1* (synovial sarcoma translocation gene on chromosome 18-like 1) gene locus associated with CpG hypermethylation at the same locus (*p* = 3.67x10^-6^). *SS18* and *SS18L1* encode transcriptional regulators and are breakpoints in chromosomal translocations in synovial sarcoma [47]. These translocations are not seen in the NCI-60 panel tumors, and whether the epigenetic signature associated with the *Alu* insertion impacts expression of this gene is unknown. So, while it is not clear at this point that *SS18L1* methylation is germane to the development of these malignancies, our ability to relate genotype and epigenetics at these sites demonstrate the value of this approach.

The large majority of statistically significant associations between insertions and cellular phenotypes appeared to involve indirect or *trans* effects that are difficult to test further. Pathway analyses suggest that many are not random, but reflect recognized, related gene sets. It may be that the indirect effects can be dissected for some insertion alleles; particularly promising may be those at loci of transcriptional regulators with definable target genes [29].

## Conclusions

In summary, we profiled LINE-1 and *Alu* insertion sites in a panel of widely used cancer cell lines, the NCI-60. We expect maps such as these will be a useful resource for experimentalists with interests in how transposable element insertions interact with genes. Our analyses show that insertion sites can be integrated with other data to develop testable hypotheses about the function of mobile DNAs in cancer.

## Methods

### NCI-60 cell lines

The National Cancer Institute-60 (NCI-60) human cancer cells are a group of 60 cell lines representing nine different types of neoplasias(breast cancer, colon cancer, CNS tumor, leukemia, lung cancer, melanoma, ovarian cancer, prostate cancer, and renal cell carcinoma) composed of 54 individual cancer cases and three pairs of cell lines (ADR and OVCAR-8; MB-435 and M14; and SNB19 and U251) with each pair originating from the same patient [48, 49]. The NCI-60 panel has been extensively characterized in a breadth of molecular and pharmacologic assay [50]. Genomic DNA was obtained directly from the NCI.

### Microarray design

A genomic tiling micorarrray was designed to cover the NCI Cancer Gene Index (disease list). A total of 6,484 RefSeq gene identifiers were extracted from the. XML file and converted to genomic coordinates corresponding to each transcript unit +/- 10 kb hg19 reference genome assembly (February 2009, GRCh37). UCSC Table Browser intervals were merged using GALAXY [51], and probes were chosen for the NimbleGen HD (2.1 M feature) array platform by the manufacturer (Roche NimbleGen, Madison, WI).

### Transposon insertion profiling by microarray (TIP-chip)

Five micrograms of genomic DNA of each cell line was digested overnight in parallel reactions using four restriction enzymes (*Ase*I, *Bsp*HI, *Hind*III, and *Xba*l). Sticky ends were ligated to annealed, partially complementary vectorette oligonucleotide adapters. Each template was aliquoted into 3 separate vectorette PCR reactions for L1Hs, *Alu*Ya5/8, and *Alu*Yb8/9 mobile DNA families. These were then labeled with Cy3-dUTP for LINE-1 and Cy5-dUTP for *Alu* and hybridized to Nimblegen genome tiling arrays according to the manufacturer’s instructions. Reference insertions are those incorporated in the Feb. 2009 assembly of the human genome (hg19, GRCh37 Genome Reference Consortium Human Reference 37, GCA_000001405.1).

### Peak recognition

Each scanned array yielded a raw .tff file, which was processed using Nimblescan v2.5 (Roche Nimblegen, Madison, WI) to give genomic coordinates and probe intensities (.gff files). A PERL script removed probes overlapping repeats to reduce noise (RepeatMasking). Nimblescan called peaks using a sliding window threshold. Peaks were ranked by the threshold of the log2 transformed ratio of red (Alu) and green (L1) channels or the reciprocal (settings: percent (p) start = 90, p step = 1, #steps = 76, width of sliding window = 1500 bp, min probes > 4, all probes > 2). The top 5,000 L1 and Alu peaks were kept for evaluation.

### Peak cut-off

Among these peaks, recovery of those corresponding to mobile DNA insertions in hg19 (reference insertions) was used as a proxy of assay performance. Reference insertion count was plotted against peaks recognized (Fig. 1c). A cut-off was imposed on the peak threshold value (p >70 for L1 and p > 60 for Alu) to include peaks up to the approximate inflection point of this curve in subsequent analyses. These threshold values were altered for outlier cell lines to reflect the curve inflection point. MYSQL was used to annotate peaks with respect to genes and known mobile DNA insertions (L1Hs, AluY, AluYa5, AluYa8, AluYb8, and AluYb9 using 1−2 kb margins). Lists of known insertions were obtained from previously published databases [14, 19, 52, 53].

### Clustering and insertion profiles

Principle component analysis (PCA) (R-package) was used to remove batch effect. All insertions were sorted by density across the cell lines and plotted as a matrix. Cell lines lacking high-frequency insertions were assessed for karyotype abnormalities manually.

### COMPARE analysis

Reference and non-reference insertions were analyzed using a COMPARE analysis [42] associating each with the CellMiner database of NCI-60 cell profiling studies. These have included DNA mutations and methylation; RNA and miRNA expression; protein expression, enzymatic activity; and drug inhibition studies. Associations for those insertions found in one cell line (singleton) were considered only for *cis* effects and were discarded from other associations due to their high false-positive rates. *P*-values for other insertions were corrected using Bonferroni multiple test correction and plotted using the start position of peak intervals to generate Manhattan plots (adaptation of Genetics Analysis Package, R-package).

### Pathway analysis

Gene loci containing candidate non-reference (polymorphic and singleton) LINE-1 and *Alu* insertions and associated gene names from RNA and protein COMPARE analysis were uploaded in batch to the MSigDb ‘Investigate Gene Sets’ from the Broad Institute Gene Set Enrichment Analysis web interface [54] (using the C2 curated gene sets). Pathways were selected if the insertion locus was part of the pathway and the p-value of the pathway was less than 10^-4^. Interactome plots were used to visualize relationships between genes in pathways using Search Tool for the Retrieval of Interacting Genes/Protein (STRING) 9.0 [55]. Plots were adapted to show the gene locus containing the insertion (yellow) and the direction of related correlations (red for positive correlations with the insertion; purple for negative correlations).

### Preferential integration sites

To investigate preferential transposable element insertion in genes implicated in oncogenesis and mouse common insertion sites, we used a hypergeometric distribution test (pHypr R-package) which controlled for genes tiled on the array. Results were plotted using the –log(*p*-value).

### Tumor-normal gene expression studies

Tumor vs normal gene expression for genes containing candidate non-reference TE insertions was assessed for each tumor type using large tumor/normal gene expression databases. Tumor gene to normal gene expression ratios were obtained using NCBI GEO2R [56]. GEO2R was used to log2 transform expression data if datasets were not in log2 formats. Value distribution of all databases was assessed for median-centering prior to evaluation. Expression values for all insertion-containing genes was plotted as a horizontal bar plot. A random sample of 1000 genes from the array were evaluated in the same manner to serve as a control set. A histogram of random gene expression values was plotted. Databases (Breast = GSE5764, Ovarian = GSE26712, omitted samples with “no evidence of disease”, Colon = GSE6988, omitted non-primary tumors, Melanoma = GSE7553, CNS = GSE4290, non-tumor used as “normal” and non-glioblastomas omitted, Prostate = GSE3325, Renal = GSE11151, non-conventional tumors omitted, NSCL = GSE19188).

### STOP gene expression in breast cancer cell lines

Expression of STOP genes containing candidate non-reference TE insertions was assessed using log2 transformed Agilent mRNA expression data [57] obtained from the CellMiner for the Breast cancer cell lines. The expression was averaged across all cell lines, sorted, and plotted as a horizontal bar plot. STOP genes tiled on the array, but without a TE insertion was plotted as well. Tumor-Normal expression for STOP genes was performed according to the methods used above in Tumor-Normal gene expression studies.

## Additional files

Additional file 1 A map of LINE-1 (L1) insertion site positions in the NCI-60 cell panel. Genomic coordinates of TIP-chip peaks are provided. Reference insertions are indicated in column D (hg19), and known polymorphic variants are indicated by a ‘Y’ in column E (Y/N, yes/no). For each cell line in columns G-BN, a ‘1’ indicates that the insertion is present, while ‘0’ indicates that the insertion is not found. (XLSX 85 kb)
Additional file 2 A map of* Alu* insertion site positions in the NCI-60 cell panel. Genomic coordinates of TIP-chip peaks are provided. Reference insertions are indicated in column D (hg19), and known polymorphic variants are indicated by a ‘Y’ in column E (Y/N, yes/no). For each cell line in columns G-BN, a ‘1’ indicates that the insertion is present, while ‘0’ indicates that the insertion is not found. (XLSX 380 kb)
Additional file 3 COMPARE analysis associating insertions with other cell characteristics. Different tabs are used for different datasets. Activity, enzyme activity measures; Decreased / Increased methylation, DNA methylation measures; Metabolome, metabolic intermediates; Drug Effect GI50, concentration for 50 % growth inhibition; Drug Effect TGI, concentration for total growth inhibition; miRNA, microRNA expression levels; RNA, mRNA expression levels; Mutations, somatically-acquired DNA mutations; Protein, protein expression. (XLSX 4049 kb)

## Abbreviations

LINE-1: Long INterspersed Element-1
NCI: National Cancer Institute
SINE: Short INterspersed Element
TIP-chip: Transposon insertion profiling by microarray
